# Notes and News

**Published:** 1892-02-27

**Authors:** 


### Notes and News.

Collected and Communicated.

We quite agree with Mr. Quennel in the remarks he makes in his reply to a letter that appeared in the *Echo*, respecting the waiting crowd of out-patients at the Westminister Hospital. This is another example of injudicious rushing into print on the part of the public. Were the grievance one possible of remedy, and yet persistently ignored by the proper authorities, after a proper representation of the case being made to them, then it would be justifiable to appeal to the powerful aid of the Press. Apparently the complainant, Mr. Batten, resorted to the medium of print at once, without enquiry, and without due consideration. The authorities of the Westminister Hospital, or indeed of any other like institution, would find it a difficult matter where it is a case of first come first served, of preventing people from securing the best chance of attention. As Mr. Quennel justly observes some fixed hour for entering must be adhered to, and the patients if refused permission to present themselves when they please in order to be before some one else, would be the first to deny Mr. Batten's right to dictate reform.

The English public is so well supplied with periodicals relating to all forms and descriptions of charity that an increase in the number might expect a somewhat indifferent welcome. In the United States they have not been so well supplied, and the *Charities Review*, which has recently been launched on the waters of public favour, bids fair both to gain and deserve a hearty welcome. It is published monthly, under the auspices of the Charity Organisation Society of New York. We have before us the fourth number of this interesting periodical, which we have perused with pleasure, and we congratulate the proprietors on the choice and treatment of the articles contained in it. The little volume introduces us to much we were unfamiliar with in the healthy activity of those zealous after good works in the Western Hemisphere. Miss Alice Miller makes us acquainted with "Hall House," an American Toynbee Hall; Mr. A. Johnson writes an able article against quasi-public charity. The underpaid workers of the female population find a hearty advocate in the Rev. Louis Banks. His book on "White Slaves: The Oppression of the Worthy Poor," is reviewed in an interesting manner by Mr. Joseph Lee. The work of the Charity Organisation is, of course, recorded; and we are placed in possession of many interesting facts relating to this and other charities. In fact, there is matter worth reading for all who are directly or indirectly interested in the well-being of our fellow-creatures.

It is not much longer than a year ago since the Duke of Clarence and Avondale opened the new buildings of the Royal Infirmary at Liverpool. The time has been long enough to judge whether the institution has fulfilled the standard of efficiency expected of it, and to show the precise amount of income required for its maintenance. That much must be expected of it is undoubted when we quote the opinion of it from the report of the Supervising Surgeon-General of the United States Hospital service, who visited it in an official capacity. He says "The Liverpool Hospital to-day is probably the best hospital in the world," and with it he compares only the Hamburg and Berlin institutions. It is obvious that with such unqualified praise that the Royal Infirmary has a great deal to live up to.

It must be gratifying to Liverpool to possess such an institution, and we can hardly think that it will demur at the comparatively small extra cost of maintenance for the new institution, which amounts to £3,000. The hospital appears to have lost a good many liberal subscribers during the year, but owing to the energy of the Committee a large number of new friends have come forward, and will, it is hoped, continue to do so. Further activity on the part of the medical staff and management is shown in the opening of the Lock Hospital, for some time closed. Interest in the Infirmary is encouraged by the opening of the chapel to the public. The little building is in itself unique and attractive. The whole of the interior is faced with harmonious-coloured tiles, which lend a bright and cleanly appearance to the building. A full description of the institution was given by our special commissioner not long since. The Infirmary is well worthy of a visit, and the visitor cannot fail to be pleasingly impressed, both with the building and internal arrangements. Where a friendly spirit combined with ability and energy prevails on the part of all in authority success cannot fail to be ensured.

The metropolitan hospitals would have little to complain of were they all to possess such a friend as the Charing Cross Hospital possesses in Mr. Passmore Edwards. There are many rich citizens by whom were they to identify themselves with some particular hospital such an opportunity to do undoubted and lasting good should be gladly hailed. So magnificent a gift as the £5,000 presented by Mr. Passmore Edwards for the establishment of a Convalescent Home for Charing Cross, is all the more admirable from the unpretentious manner in which the gift is made. Mr. Edwards expresses the hope that his gift will rather encourage than deter others from exercising their generosity. He clearly sees the tendency which large donations and legacies have of diminishing the smaller but necessary and valuable subscriptions. The number of in-patients at the Charing Cross Hospital in 1891 was in excess of previous years, and an increased liberality therefore is called for at the hands of the public.

The annual meeting of the Governors of the Metropolitan Hospital was held on Wednesday the 17th, at the institution, Kingsland Road, under the presidency of Mr. Joseph Fry. Mr. C. H. Byers, the Secretary, read the annual report, which showed that the work of the charity had largely increased during the last twelve months. The number of the in-patients had advanced from 709 to 880, the out-patients from 14,104 to 15,358, and the total out-patient attendances from 66,506 to 69,196. The receipts showed a decrease of £550, and the expenditure an increase of nearly £400, the former being due to the small amount received from legacies £798, as compared with £2,140 in 1890, and the latter to the greater number of patients treated. The grants to the institution from both the Hospital Sunday and Saturday Funds were largely in excess of those of the previous year. Lord Sandhurst, Chairman of the Royal Commission on Hospitals, had promised to preside at the festival dinner on behalf of the hospital on June 30th next, at the Hotel Métropole. The Chairman, in proposing the adoption of the report, which was agreed to, made an earnest appeal to the public for increased and liberal support to the institution.

